# Anemia and associated factors among patients admitted with metabolic syndromes at Worabe Comprehensive Specialized Hospital, Southern Ethiopia: A cross-sectional study

**DOI:** 10.1371/journal.pone.0266089

**Published:** 2022-04-04

**Authors:** Abebe Timerga, Kassahun Haile, Samuel Dessu

**Affiliations:** 1 Department of Biomedical Science, School of Medicine, College of Medicine and Health Sciences, Wolkite University, Wolkite, Southern Ethiopia; 2 Department of Medical Laboratory Science, College of Medicine and Health Sciences, Wolkite University, Wolkite, Southern Ethiopia; 3 Department of Public Health, College of Medicine and Health Sciences, Wolkite University, Wolkite, Southern Ethiopia; Suez Canal University Faculty of Medicine, EGYPT

## Abstract

**Background:**

Anemia is a condition in which the number of red blood cells is inadequate to meet the physiologic needs of the human body oxygen and it is highly prevalent among individuals with metabolic syndromes as a complication in turn speed up the progression and the number of adverse outcomes unless the proper measure is undertaken. Determination of anemia may play a major role in the management and early aversion of complications in an admitted patient with metabolic syndromes. Therefore, this study aimed to determine anemia and its associated factors among patients with admitted metabolic syndromes at Worabe Comprehensive Specialized hospital, Southern Ethiopia from March 1 to May 30, 2021.

**Methods and materials:**

An institutional-based cross-sectional study design was conducted on 324 adult patients admitted with metabolic syndromes. Structured questionnaires through face-to-face interviews and participants’ medical records were used to collect information on determinants related to anemia. A blood sample was collected to determine hematological parameters, biochemical profile, and blood film preparation. Data were analyzed by SPSS version 22. Binary logistic regression analyses were done to identify factors associated with anemia. The p-value was set at <0.05 with a 95% confidence interval of the adjusted odds ratio.

**Results:**

A total of 324 admitted adult patients with metabolic syndromes were involved. The overall prevalence of anemia among study participants was 25.3% (95%CI: 20.7, 30.2), of which 52.4% had moderate anemia and 56% had microcytic types of anemia. Being alcoholic (AOR = 6.25, 95% CI: (3.05, 12.82)), obese (AOR = 3.34, 95% CI: (1.02, 11.21)), dyslipidemic (AOR = 2.06, 95% CI: (1.02, 4.17)), and diabetic (AOR = 2.61, 95%CI: (1.31, 5.21)) were significantly associated with anemia.

**Conclusion:**

The prevalence of anemia among patients admitted with metabolic syndrome observed in this study was a moderate public health problem. Taking alcohol, being dyslipidemic, obese and diabetic was significantly associated with anemia. The finding of this study should be taken into consideration to implement preventive interventions on identified factors in study percipients. Taking fruit and vegetable, and promoting physical exercise, routine determination of fasting blood glucose and hemoglobin level in adult admitted metabolic syndromes patients were recommended to minimize the emergence of anemia.

## Introduction

Metabolic syndrome(MetS) is a set of interconnected and undesirable metabolic, physiologic, biochemical, and clinical conditions, resulted in advert health outcome; it is also associated with the increased prevalence of obesity, atherogenic dyslipidemia, high blood pressure, sedentary way of life, population growth, and urbanization [1, 2]. Metabolic syndromes are also known as insulin resistance syndrome which encompasses and characterizes a group of clinical and laboratory diagnostic test abnormalities, that are associated with the increased risk of cardiovascular diseases, diabetes mellitus, and related complications [1]. In sub-Saharan Africa, the burden of MetS is projected to surpass infectious diseases by 2030 and a cross-sectional study conducted among working adults in Ethiopia revealed that the overall prevalence of MetS was 12.5% and 17.9% according to Adult treatment panel III (ATP III) and International diabetic federation (IDF) definitions, respectively [3].

Anemia is a condition in which the number of red blood cells (RBCs) is inadequate to meet the physiologic needs of the human body oxygen and it is prevalent among individuals with certain chronic conditions, including chronic diseases, human immunodeficiency virus, rheumatoid arthritis, congestive heart failure, and cancer [4, 5]. Currently, MetS considered as a major cause of death, due to the increased risk of developing complications related with cardiovascular diseases including anemia, which contribute to the high burden of death due to increased derangement of lipids [6, 7].

Factors that contributed to the onset of anemia in MetS patients include systemic inflammation, inhibition of erythropoietin release, and damage to the renal parenchyma, symptomatic autonomic neuropathy, and secondary complications [8, 9]. Hyperglycaemia, which is the earliest onset due to MetS, has a direct relationship with the development of inflammatory conditions by expressing pro-inflammatory cytokines like interleukin-6 (IL-6), tumor necrotizing factor-*α* (TNF*α*), and necrotizing factor (NF) [10, 11]. The elevation of those cytokines has an important role in the development of insulin resistance and induces the appearance of cardiovascular complications in admitted MetS patients including anemia [12, 13]. By increasing especially IL-6, the anti-erythropoietin effect occurs, since this cytokine decreases the sensitivity of receptors to erythropoietin and also enhances apoptosis of developing erythrocytes [14]. Moreover, the number of circulating erythrocytes decrease and causing a reduction of circulating hemoglobin [11, 15]. Some nutritional deficiencies’ like cyanocobalamine and folate can cause another type of anemia [11]. In addition medications like metformin, angiotensin-converting enzyme inhibitor and angiotensin receptor blockers can cause anemia by interfering with nutritional absorption [16, 17].

Furthermore, in a late stage of the disease renal complication may arise, which further complicates the renal production of erythropoietin [18]. Finally, the capacity of blood to transport oxygen to tissues is reduced due to the decrement of hemoglobin, RBC count, and hematocrit. In turn, it causes cardiovascular and end-stage renal diseases that enhance the progression of disease morbidity and mortality in MetS patients [19, 20]. Anemia is not a disease but a manifestation of a different disease that ameliorates one’s quality of life [12, 21].

Even though knowing the status of anemia and its associated factors in admitted MetS patients is significant, the problems were not well studied in our setup and few with conflicting information are available. So, conducting this study is crucial to improve early diagnosis, prevention of complications, and enhance the quality of life of MetS patients. Therefore, this study was conducted to assess the burden of anemia and its associated factors among admitted MetS patients in Worabe Comprehensive Specialized hospital.

## Materials and methods

### Study setting, period, and design

The study was conducted among patients admitted with metabolic syndromes at Worabe Comprehensive Specialized hospital from March 1^st^ to May 30^th^, 2021 which is located in Silte zone, SNNPR region of Ethiopia. A facility-based cross-sectional study design was employed to carry out the project.

### Populations of the study

The source populations comprised of all patients who were admitted to Worabe Comprehensive Specialized hospital with MetS and the study population comprised of all the selected patients with MetS during the study period. All admitted adult patients (≥18 years) who presented with three or more of the national cholesterol education adult treatment panel III (NCEP-ATP III) major criterias were enrolled in the study and those patients who don’t fulfill national cholesterol education adult treatment panel III (NCEP-ATP III) major criteria (triglyceride level <150 mg/dL, HDL-C >40 mg/dL in males, >50 mg/dL in females: systolic BP <130 mmHg or diastolic BP <85 mmHg, fasting glucose <110 mg/dL [2] and MetS patients with blood cell malignancy, mothers with pregnancy, patient with chronic renal disease, patients who were critically ill and those who have bleeding history during data collection period were excluded from the study.

### Sample size and sampling techniques

A single population proportion formula was used to calculate the minimum sample size required by considering the following assumptions: expected frequency (the prevalence of anemia among admitted metabolic syndrome patients = 26% [22], 95% confidence level, 5% degree of precision, and non-response rate of 10% which gives 324. All consecutively identified patients admitted with metabolic syndrome and who fulfilled the inclusion criteria were enrolled in the study.

### Data collection methods and procedure

Pre-tested interviewer-administered structured questionnaires were used to collect socio-demographic, clinical, and behavior-related data. Two BSc nurses with previous experience in data collection and procedures were recruited for data collection. Two days training was given to the data collectors before the data collection period to familiarize them with the objective of the study. Continuous supervision was provided by the supervisor and principal investigator throughout the data collection periods.

### Anthropometry and blood pressure measurements

Bodyweight and height were measured according to the WHO guideline manual. Bodyweight was measured to the nearest 0.1kg using portable weighing scales. Body mass index (BMI) was calculated as the weight in kilograms (kg) divided by the square of the height in meters (m^2^) [6]. Waist circumference (WC) was measured at the midpoint between the lower margin of the least palpable rib and the top of the hip or minimal waist using stretch-resistant tape. The cut-off point for waist circumference ≥ 102cm for men and ≥ 88 cm for women were used to indicate central obesity. Hip circumference was measured at the widest part of the buttocks. Blood pressure was taken using a mercury sphygmomanometer from the left arm using a mercury-based sphygmomanometer after the subjects had rested for more than 10 minutes. For those study participants with systolic blood pressure (SBP) ≥140 mmHg and diastolic blood pressure (DBP) ≥90 mmHg, the blood pressure was measured again and the average value was taken [1].

### Sample collection, handling, and laboratory procedure

After obtaining written consent from the study participant in the study, the vein in which we took blood was disinfected with 70% alcohol and a tourniquet was applied. Then, 5 ml of fasting blood was collected from each study participant by a trained health professional using a sterile technique by vacutainer tube method in two different test tubes. After the required amount of blood had been collected, the tourniquet was released and enforcement of the blood count was performed to determine hematological profile determination in patients with MetS. Hematological parameters were determined by the Sysmex XP-300 hematology analyser (Sysmex XP-300, Diagnato, Mumbai, India). The blood sample was also used for the separation of serum for biochemical measurements. To do this procedure; the drawn sample was left for 30 minutes, and then serum was separated from the collected blood sample by centrifugation at 3000 rpm for 10 minutes using a Rotanta 960 centrifuge in a thermo-stable condition (Rotanta 960 tuttlingen, Germany). Then, serum was taken and stored at −20°C in the central laboratory till the time of biochemical analysis was done. Fasting glucose and lipid profile content were determined using standard principles and procedures according to the manufacturer’s instruction with an ABX Pentra 400 automated chemistry analyser (Horiba ABX SAS, Montpellier, France).

### Data quality management and data analysis

Before data analysis took place, all data and specimen collectors were investigated to control any kind of procedures and processes that may affect the result. And all the data from the questionnaires were checked for completeness and clarity. After checking the data were coded, cleaned, and entered to EpiDta3.1 and exported to SPSS version 22 for further analysis. Descriptive statistics like frequency tables, mean, median, Correlation analysis and standard deviation were computed. After cross-checking, cleaning was made to avoid missing values, and inconsistencies were removed before doing the analysis. Both bivariable and multivariable logistic regression analyses were computed to identify associations between dependent and independent variables. Crude and adjusted odds ratio with their 95% CI was computed to determine the strength and presence of association. Variables having a p-value <0.25 in the bivariable analysis were a candidate for multivariable logistic regression. Factors significantly associated with anemia in the final model were identified at p-value <0.05 with 95% CI of AOR. Appropriateness of the analysis model was checked using the Hosmer-Lemeshow test. The results were presented by using frequency tables, figures, and texts.

### Variables

The dependent variable was being anemic and the independent variables were sociodemographic factors, behavioral characteristics, medication-related characteristics, and anthropometric variables including; height, weight, body mass index, and waist circumference were included.

In this study, patients who presented with three or more of the following criteria proposed by the National Cholesterol Education Program (NCEP-ATP III) was classified as having metabolic syndrome as increased waist circumference (>88cm women and >102cm for men), elevated serum triglycerides (≥150mg/dL) or Decreased HDL cholesterol (<40mg/dL men and <50mg/dL for women), raised blood pressure: systolic BP≥140 or diastolic BP≥90 mm Hg and 4) and raised fasting plasma glucose≥110mg/dL [2].

Anemia in patients with MetS was defined based on World health organization criteria (WHO) as a Hgb concentration <13 g/dl for males and < 12 g/dl for females [23]. Anemia also further classified into mild anemic (female: 11–11.9 g/dl; male: 11–12.9 g/dl), moderate anemic (8–10.9 g/dl) and severe anemic (< 8 g/ dl) [22]. Also anemia type was classified as microcytic when MCV < 80 fl, macrocytic: MCV >100 fl and normocytic:MCV 80-100fl [4]. In addition, thin blood films form peripheral morphology were prepared, air-dried, labelled and then stained by wrights stain to evaluate RBC morphology of anemic patients. Physical activity implies participants who did not participate in moderate daily physical activities such as walking, cycling, or doing that had important benefits for health, or those participants who were not involved recommended 30–60 minutes of aerobic exercise 3–4 times per week to promote cardiovascular fitness [24].

A respondent who drank three to four units for males and two to three units for females daily at the start of the study was defined as an alcohol user during the period of data collection [25].

### Ethical approval and consent to participate

Ethical clearance was obtained from Wolkite University IRB/committee; concerned administrative offices were communicated with a formal letter. After getting permission, written consent was obtained from each study participant. Participants were informed about the purpose and procedure of the study. Confidentiality of the information was assured and the privacy of the participant was maintained by keeping their information anonymous. Based on laboratory results, study participants were referred to the physicians for further care and treatment.

## Result

Among a total of 324 admitted MetS patients, 40.1% (n = 130) females, and 59.9% (194) males were enrolled in the current study with a response rate of 100%; The median age of the respondents was 46 years (IQR: 34, 55). The majority of the study participants 46.3% (150) were in ≥50years of age groups. About 166(51.2%), 159(49.1%), and 154(47.5%) participants were urban dwellers, merchants, and physically active, respectively. Further more, the mean waist circumference of the patients was 98.75±8.46 cm with range of 81–129 cm. When we come to BMI level, about one-third 121(37.3%) of participants were overweight, and 179(55.2%), 152(46.9%) were hypertensive and dyslipidemic, respectively. The mean ±standard deviation (SD) of the FBS and BMI was 118±21.85 and 26.40±3.29 respectively. One hundred fifty (46.3%) of study participants presented with documented records of at least one of the MetS related renal complications (Table 1).

**Table 1:** Distribution of socio-demographic and clinical characteristics of study participants (n = 324).

Variables	Categories	Frequency (%)	Anemia	
			Yes (%)	No (%)
Age (years)	>= 50	150(46.3)	51 (34)	99(66)
	25–49	114(35.2)	21(18.4)	93(81.6)
	<= 25	60(18.5)	10(16.7)	50(83.3)
Sex	Female	130(40.1)	40(30.8)	90(69.2)
	Male	194(59.9)	42(21.6)	152(78.4)
Residence	Rural	158(48.8)	46(29.1)	112(70.9)
	Urban	166(51.2)	36(21.7)	130(78.3)
Occupational status	Farmer	58(17.9)	15(25.9)	43(74.1)
	Daily labourer	21(6.5)	4(19)	17(81)
	Merchant	159(49.1)	39(24.5)	120(75.5)
	civil servant	86(26.5)	24(27.9)	62(72.1)
Alcohol consumption	Yes	143(44.1)	64(44.8)	79(55.2)
	No	181(55.9)	18(9.9)	163(90.1)
Physical activity	No	170(52.5)	33(19.4)	137(80.6)
	Yes	154(47.5)	49(31.8)	105(68.2)
Medication	Yes	150(46.3)	46(30.7)	104(69.3)
	No	174(53.7)	36(20.7)	138(79.3)
Body mass index status (Kg/m^2^)	>= 30	67(20.7)	38(56.7)	29(43.3)
	25–29.9	121(37.3)	23(19)	98(81)
	18.5–24.9	116(35.8)	15(12.9)	101(87.1)
	<18.5	20(6.2)	6(30)	14(70)
Dyslipidemia	Yes	152(46.9)	62(36)	110(64)
	No	172(53.1)	20(13.2)	132(86.8)
Renal complications	Yes	150(46.3)	41(32.8)	84(67.2)
	No	174(53.7)	41(20.6)	158(79.4)
Hypertension	Yes	179(55.2)	53(29.6)	126(70.4)
	No	145(44.8)	29(20)	116(80)
FBS	≥110 mg/dL	164(50.6)	56(34.1)	108(65.9)
	<110 mg/dL	160(49.4)	26(16.3)	134(83.8)

Concerning the overall burden of anemia; 82(25.3%(95% CI: (20.7, 30.2))) of patients admitted with MetS was anemic from those anemic patients 39(47.6%) were mild, 43(52.4%) were moderately anemic and no severe anemia was identified. The mean (± SD) of hemoglobin concentration of the study participants was 13.93±2.53 mg/dl. Out of the anemic study participants 17(20.8%), 46(56%), and 19(23.2%) were normocytic, microcytic, and macrocytic types of anemia, respectively.

### Correlation analysis between hemoglobin level and clinical predictor variables of MetS

The correlation analysis between hemoglobin level and independent clinical predictors of MetS was assessed. Based on the assessment, level of haemoglobin level showed weak positive correlation with SBP (r = 0.11, p = 0.048) and moderately strong positive correlation with triglyceride levels (TG) and fasting blood sugar (FBS) (r = 0.341, p = 0.001) and(r = 0.412, p = 0.001) respectively. Low-density lipoprotein cholesterol (LDL-c) and high-density lipoprotein (HDL-C) of MetS patients had a significantly strong positive and negative correlation with hemoglobin level (r = -0.462, P = 0.000) and (r = -0.432, p = 0.021) respectively (Table 2).

**Table 2:** Correlation analysis of hemoglobin and clinical factors.

Clinical predictor variables	Mean±SD	Categories	Frequency (%)	Haemoglobin level	
				r	p-value
SBP	154.24±13.13	SBP<140	145(44.8)	0.11	0.048
		SBP≥140	179(55.2)		
DBP	97.24±6.87	DBP<90	121(37.3)	0.08	0.120
		DBP≥90	203(62.7)		
TG	142.5±26.3	<150mg/dl	178(54.9)	0.341	0.001
		>= 150mg/dl	146(45.1)		
LDL-C	100.66±10.22	<100mg/dl	181(55.9)	0.462	<0.001
		>= 100mg/dl	143(44.1)		
HDL-C	47.67±8.29	<40mg/dl	169(52.3)	-0.432	0.021
		>= 40mg/dl	154(47.7)		
FBS	118±21.85	<110mg/dl	160(49.4)	0.412	0.001
		>= 110mg/dl	164(50.6)		

### Factors associated with anemia among admitted MetS patients

Variable associated with anemia in bivariate analysis were showed that, higher age (COR (95% CI) = 2.57(1.21,5.49), being a female gender (COR (95% CI) = 1.69(0.97,2.66), alcohol consumers (COR (95% CI) = 7.33(4.07,13.20), study subjects with medication (COR (95% CI) = 1.69(1.02,2.80), higher BMI levels (COR (95% CI) = 3.05(1.04,8.92), being physically inactive, dyslipidemic, diabetic and hypertensive were candidate variables to be tested for the association within multivariable logistic analysis by considering p-vale<0.25.

After adjusting for other confounding variables: alcoholic patients were ∼ 6 times more likely to develop anemia (AOR: 6.25, 95% CI: **(3.05, 12.82))** than non-alcoholic groups. MetS patients with dyslipidemia were higher odds of having anemia (AOR: 2.06, 95% CI: **(1.02, 4.17))** than MetS patients with no dyslipidemia. Patients with BMI >= 30 Kg/m^2^ were more likely to develop anemia (AOR: 3.34, 95% CI: **(1.02, 11.21))** compared to their counterparts, and those MetS patients with diabetes were at higher odds of having anemia (AOR: 2.61, 95% CI: **(1.31, 5.21))** compared to non-diabetic MetS patients (Table 3).

**Table 3:** Bivariable and multivariable analysis of factors associated with anemia among admitted MetS patients attending Worabe Comprehensive Specialized hospital, Southern Ethiopia from March 1 to May 30, 2021.

Variables	Categories	Anemia (%)		p-value	COR (95% CI)	AOR(95%CI)
		Yes (%)	No (%)			
Age	>= 50	51(34)	99(66)	0.014	2.57(1.21,5.49)*	0.82(0.28,2.42)
	25–49	21(18.4)	93(81.6)	0.77	1.12(0.49,2.58)	1.85(0.69,4.96)
	<= 25	10(16.7)	50(83.3)	1	1	1
Sex	Female	40(30.8)	90(69.2)	0.065	1.69(0.97,2.66)*	2.46(0.66,9.1
	Male	42(21.6)	152(78.4)	1	1	1
Residence	Rural	46(29.1)	112(70.9)	0.125	0.67(0.40,1.11)*	0.18(0.05,0.72)
	Urban	36(21.7)	130(78.3)	1	1	1
Occupational status	Farmer	15(25.9)	43(74.1)	0.411	1.64(0.50,5.39)	
	Daily labourer	4(19)	17(81)	0.581	1.38(0.43,4.35)	
	Merchant	39(24.5)	120(75.5)	0.533	1.48(0.43,5.11)	
	civil servant	24(27.9)	62(72.1)		1	
Alcohol consumption	Yes	64(44.8)	79(55.2)	<0.001	7.33(4.07,13.20)*	6.25(3.05,12.82)**
	No	18(9.9)	163(90.1)	1	1	1
Physical activity	No	33(19.4)	137(80.6)	0.011	1.93(1.16,3.22)*	0.73(0.37,1.46)
	Yes	49(31.8)	105(68.2)	1	1	
Medication	Yes	46(30.7)	104(69.3)	0.040	1.69(1.02,2.80)*	0.55(0.28.1.07)
	No	36(20.7)	138(79.3)	1	1	
BMI (Kg/m^2^)	>= 30	38(56.7)	29(43.3)	0.001	3.05(1.04,8.92)*	3.34(1.02,11.21)**
	25–29.9	23(19)	98(81)	0.265	0.458(0.19,1.57)	0.34(0.09,1.24)
	18.5–24.9	15(12.9)	101(87.1)	0,059	0.347(0.11,1.04)	2.22(0.61,8.04)
	<18.5	6(30)	14(70)	1	1	1
Dyslipidemia	Yes	62(36)	110(64)	<0.001	3.72(2.11,6.53)*	2.06(1.02,4.17)**
	No	20(13.2)	132(86.8)	1	1	1
renal complications	Yes	41(32.8)	84(67.2)	0.015	0.532(0.32,0.88)*	0.80(0.41,1.56)
	No	41(20.6)	158(79.4)	1	1	1
Hypertension	Yes	53(29.6)	126(70.4)	0.049	1.68(1.00,2.82)*	2.46(0.24.4.89)
	No	29(20)	116(80)	1	1	1
FBS	≥110 mg/dL	56(34.1)	108(65.9)	<0.001	2.67(1.57,4.53)*	2.61(1.31,5.21)**
	<110 mg/dL	26(16.3)	134(83.8)	1	1	1

## Discussion

Metabolic syndromes and other cardiovascular complications have played great attention as a potential clinical condition predicting the development of complications like anemia in various ways and in turn compromising the management of the disease itself and its progression [14, 26, 27].

The current study aimed to determine the prevalence and associated factors of anemia among admitted MetS patients in southern Ethiopia. The overall prevalence of anemia among admitted MetS patients was 25.3%. According to the WHO classification of the public health importance of anemia [23] anemia prevalence among admitted MetS patients in this study indicated a moderate public health problem. The findings of our study is consistent to the finding from studies conducted in eastern Ethiopia, the University of Bagdad, and India where 34.8%, 26%, 26% of study participants were found to be anemic respectively [4, 5, 22]. Also a similar study was done Shebin EloKom Teaching Hospital, Shebin EloKom, Menoufia Governorate, Egypt where the magnitude of anemia was found to be 65% [11]. The variation attributed might be the difference in socio-demographic and behavioral characteristics and the inclusion of patients with their chronic complications and in some of the studies there is inclusion of participants with other co-morbidities too [5, 22].

However, the result was higher when compared to the study done in Debre Berhan Referral Hospital, North-East Ethiopia on diabetic patients and Gandhinagar, Gujarat, India among type-2 diabetes mellitus patients and Aseer Province, Kingdom of Saudi Arabia among patients with a chronic disease where the prevalence of anemia was 20.1%, 18% and 20.83% respectively [19, 28, 29]. The variation might be attributed to the majority of participants in this were diabetics without inherent complications in which, patients who have insulin resistance with a decrease in excretion of substances due to the reduced effects of insulin action, in turn, a mild effect on erythropoietin. In addition, the difference in variability of the study participants may contribute to magnitude variation.

In this study among the anemic study participants 20.8%, 56%, and 23.2% were normocytic, microcytic, and macrocytic types of anemia, respectively. Considering the severity of anemia, moderate anemia (52.4%) was the predominant type of anemia among admitted MetS patients followed by mild anemia (47.6%) in this study. This might be due to systemic inflammation, inhibition of erythropoietin release, rise in pro-inflammatory cytokines, and damage to the renal parenchyma [30].

This study showed that study subjects with alcoholics were found to increase the odds of anemia by nearly 6 folds (AOR: 6.25, 95% CI: **(3.05, 12.82**)) than non-alcoholics. This was in line with studies conducted at the University of Washington Medical Center, Seattle, and New York. The possible justification could be, as subjects got more alcoholic there will be generalized suppression of blood cell production and the production of structurally abnormal blood cell precursors that cannot mature and function. Alcoholic MetS frequently have defective red blood cells that are destroyed before achieving maturity which results in causing anemia. In addition, alcohol can interfere with the proper absorption of iron into the hemoglobin molecule. Furthermore, alcohol use can lead to either iron deficiency or excessively high levels of iron in the body especially in the liver concomitantly causing transferrin loss that enhances absorption of iron. Since iron is essential to RBC functioning, iron deficiency, which is commonly caused by anemia [31–33].

Also, subjects with obesity (higher BMI) and dyslipidemia have significantly associated with anemia in which the likely hood of developing anemia was **3.34** and **2.06** higher in participants when compared with counterparts respectively. This was in line with studies conducted at Batman State Hospital, Batman, Turkey, Al-Kindy College of Medicine, University of Baghdad, İzmir, Turkey, and Guangdong Province, China [4, 13, 20, 26]. The association might be dues to the fact that obesity and abnormal lipid stores down-regulate the ferroportin-1 exporter by hepcidin since obesity and dyslipidemia are associated with chronic, low grade, systemic inflammation by activating different cytokines which in turn up-regulates the transcription of protein hepcidin that results in sequestration of iron within entrecote, hepatocytes, and iron storing macrophages that will reduce iron bioavailability [34, 35].

Another factor that significantly associated with anemia is type-2 diabetes (FBS≥110 mg/dL); anemia (AOR: 2.61, 95%CI: **(1.31, 5.21))** this was in line with studies conducted in North-East Ethiopia, University of Northwestern Rio, Brazil, and Haramaya University, Harar, Ethiopia [5, 19, 36]. Since those patients with raised glucose affect precursors of erythrocyte in the bone marrow or inhibition of erythropoietin release from the renal system. Especially in neglected and complicated diabetes glucose toxicity leads blood cells to oxidative stress causing disturbances in the red blood cells function. Additional factors which have been implicated in the development of anemia in hyperglycaemic patient include; systemic inflammatory, damage to renal architecture and its effects on bone marrow [11, 21].

### Limitations of the study

Although the necessary opportunities were made to minimize the possible limitations of this study, the interpretation of the result should be considered in light of the following limitations. There might be recall bias since study participants were asked for situations that happened in the past. Due to the nature of the study design, the cause/effect relationship was not determined. Furthermore, this study might be among a few which tried to determine anemia in admitted MetS patients in developing country like Ethiopia.

## Conclusion

Hematological disorders especially, anemia is common and highly prevalent in admitted MetS patients and may be related to increased adverse complications. In particular, those patients having, alcohol consumers, and patients with high BMI levels are at increased risk of developing anemia. Prevention of high blood glucose level, dyslipidemia, and obesity particularly; by doing moderate physical exercise and by eating a healthy diet, when possible, strict follow-up and regular screening for anemia in all MetS patients and control of glucose level are paramount importance to avert having anemia. The most important management of these derangements can best be achieved by averting the underlying pathophysiologic events.

## Supporting information

S1 Dataset (SAV)
